# Optimization of Phenolic Antioxidant Extraction from Wuweizi (*Schisandra chinensis*) Pulp Using Random-Centroid Optimazation Methodology

**DOI:** 10.3390/ijms12096255

**Published:** 2011-09-23

**Authors:** Xia Wu, Xiong Yu, Hao Jing

**Affiliations:** 1 College of Food Science and Nutritional Engineering, China Agricultural University, Beijing 100083, China; E-Mail: lulmowx@hotmail.com; 2 College of Animal Science, Xinjiang Agricultural University, Urumqi 830052, China

**Keywords:** Wuweizi, pulp, polyphenols, extraction, random-centroid optimization

## Abstract

The extraction optimization and composition analysis of polyphenols in the fresh pulp of Wuweizi (*Schisandra chinensis*) have been investigated in this study. The extraction process of polyphenols from Wuweizi pulp was optimized using Random-Centroid Optimization (RCO) methodology. Six factors including liquid and solid ratio, ethanol concentration, pH, temperature, heating time and extraction times, and three extraction targets of polyphenol content, antioxidant activity and extract yield were considered in the RCO program. Three sets of optimum proposed factor values were obtained corresponding to three extraction targets respectively. The set of optimum proposed factor values for polyphenol extraction given was chosen in further experiments as following: liquid and solid ratio (v/w) 8, ethanol 67.3% (v/v), initial pH 1.75, temperature 55 °C for 4 h and extraction repeated for 4 times. The Wuweizi polyphenol extract (WPE) was obtained with a yield of 16.37 mg/g and composition of polyphenols 1.847 mg/g, anthocyanins 0.179 mg/g, sugar 9.573 mg/g and protein 0.327 mg/g. The WPE demonstrated high scavenging activities against DPPH radicals.

## 1. Introduction

Wuweizi (*Schisandra chinensis* (Turcz.) Baill.) (“five flavored berry” in Chinese) is a berry-like fruit, mainly grown in north-eastern China, including Heilongjiang, Jilin, Liaoning provinces [1]. The growing area for Wuweizi in north-eastern China is about 10,000 hectares as documented in 2010 and the annual production increased from 3200 ton in 2007 [2] to 13,900 ton in 2010 due to the high market demand. Wuweizi has been used as a nutritional and functional ingredient in foods, such as wine, beer, beverages, yoghourt, jam, fruitcake and other products [3]. Wuweizi is also used in Chinese traditional medicine [4]. Recently, some new bioactivities of Wuweizi have been reported, such as hepatoprotection, anti-carcinogenicity, anti-obesity, and anti-diabetes [5].

There were a number of bioactive components identified in Wuweizi, such as lignans, organic acids (citric, malic, fumaric and tartaric acid), and essential oils [5]. Wuweizi has high content of lignans, which mainly exist in seeds (4.1~19.2% of whole fruit) [1]. The lignans have been considered as the primary constituents for Wuweizi’s antioxidant, hepatoprotective, immuo-modulating, anti-inflammatory and anticancer effects [6]. Compared to the intensive studies on lignans, polyphenols in Wuweizi pulp, especially anthocyanins have not received much attention. There were a few of reports about anthocyanins extracted from Wuweizi. Yang *et al.* reported that the anthocyanin content was about 0.085% [7], while Kim *et al.* reported 0.2% anthocyanins for fresh whole fruit weight of Omija (*Schisandra chinensis*) grown in Korean [8].

The extraction of polyphenols, especially anthocyanins, was influenced by many factors such as type of solvent, solvent and water ratio, pH, temperature and extraction times [9]. Some optimization methodology such as response surface methodology (RSM) and orthogonal experimental design (OED) have been applied for optimizing polyphenol extraction. Both RSM and OED were run based on single-factor tests (SFT) with only one extraction target, resulting in different sets of factor values before finding a set of optimized factor values. There are some reports about using SFT and OED to optimize the extraction of “colors” from Wuweizi whole fruits, but the optimized factor values were different [10,11]. The random-centroid optimization (RCO) methodology was developed by Dou and Nakai of the University of British Columbia in 1993 and has been successfully used in optimization of site-directed mutagenesis, lysozyme-dextran complex preparation conditions, and food formulation [12–15]. RCO program is able to input all potential factors at the same time in a random search and provides several sets of proposed factor values. Each set of proposed factor values is followed by experiment, and the obtained extract is analyzed for extraction target value (one extraction target value) or values (more than one extraction target). The extraction target value is entered into RCO program for a centroid search, which provides fewer new sets of proposed factor values for further test experiments. Finally, RCO program proposes a set of optimum factor values. RCO methodology could propose simultaneously several sets of optimum factor values for several extraction targets with a minimum of one cycle (consists of random search, centroid search and mapping). The values of factors could be adjusted and allow the investigator to incorporate reference values and preliminary results.

The aim of the present study was to optimize extraction process of Wuweizi pulp polyphenols using RCO methodology. The polyphenol content, antioxidant activity and extract yield were evaluated as three extraction targets for optimizing extraction process. The composition analysis and antioxidant activity of Wuweizi polyphenol extract were also performed in this study.

## 2. Results and Discussion

RCO program ran a random search based on the six factors with a selected range of empirical values, and presented twelve sets of factor values proposed for optimum extraction process (Table 1). Each set of proposed factor values (Table 1) was followed by experiment, and the obtained extract was analyzed for polyphenol content, DPPH radical-scavenging activity and extract yield, respectively. The measured values are presented in Table 2, which were input into RCO program for a further optimization search. After a centroid search, four new sets of factor values were proposed for each of three extraction targets, *i.e.*, polyphenol content, DPPH radical-scavenging activity and extract yield, respectively, for further experiments (Table 3). The four extracts were obtained by following the four new sets of factor values proposed for each of the three extraction targets, and analyzed for corresponding extraction target values (Table 4).

**Figure 1:**
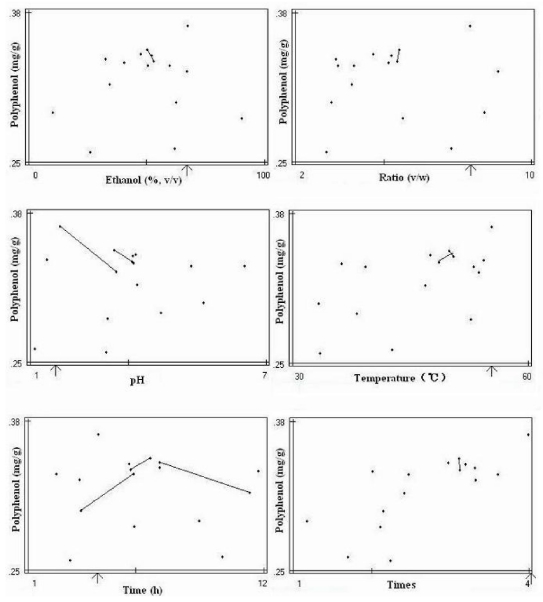
Optimization mapping for polyphenol as extraction target.

The final optimization mapping provided an optimum set of factor values for each extraction target, presented in the form of mapping. The total six maps with six optimum factor values were presented for optimum extraction conditions of polyphenol (Figure 1). The arrow in each map points to the optimum value of each factor. The similar mapping for optimum extraction conditions of DPPH radical-scavenging activity and extract yield were also obtained (data not shown). The mappings for the pH and heating time factors showed more trend lines toward optimum values, while the mappings for other factors showed less trend lines with undefined directions. These results indicated that the pH and heating time were important factors, which affected greatly extraction process. The final three sets of optimum factor values for three extraction targets were obtained from the mappings (Table 5). Each set of proposed optimum factor values was followed by experiment, and the three extracts corresponding to three extraction targets were obtained and analyzed for polyphenol content, DPPH radical-scavenging activity and extract yield, respectively (Table 6).

The compositions of the three extracts varied, because they were obtained by different optimum extraction processes with different extraction targets. The extract obtained from optimum extraction process for polyphenol content had high polyphenol content with reasonable yield; the extract obtained from optimum extraction process for extract yield had high yield with reasonable polyphenol content; the extract obtained from optimum extraction process for DPPH scavenging rate did not demonstrate high DPPH scavenging rate, and similar DPPH scavenging rates were observed for all three extracts (Table 6). The three extracts were further analyzed for their compositions. The extract obtained from optimum extraction process for polyphenol content extract had also high sugar and protein contents along with reasonable anthocyanin content in comparison with other two extracts (Table 7).

The six factors of ethanol concentration, pH, liquid and solid ratio, temperature, heating time and extraction times were considered to influence the polyphenol extraction. The value ranges of the six factors were chosen and input for RCO methodology, based on reference literature and our preliminary experiments [16]. Water and different ethanol concentrations had been used as extraction solutions for polyphenol [8,10,11], so the value range of ethanol concentration was set at 0–100% for RCO methodology. Liquid and solid ratios of 4, 7 and 10 have been reported for polyphenol extraction [16–18], so the value range of liquid and solid ratio was set at 2–10. Our preliminary experiment showed that the polyphenol of Wuweizi is stable at a temperature under 60 °C, so the value range of temperature was set at 30–60 °C. The heating time and extraction times varied among the studies. For example, the heating time from 1 h to 12 h and extraction times from 1 to 4 were used in polyphenol extraction of grape, seaweed and blueberry [16,19,20], so the value ranges of heating time and extraction times were set at 1–12 h and 1–4 times, respectively. Polyphenol is stable at pH 3.0–7.0. It could be readily oxidized by polyphenol oxidase to quinine structure when pH > 7.0 [21]. 0.05–0.1% HCl have been used to keep the acidic environment during polyphenol extraction [22,23]. Our preliminary data showed that the pH was about 3.0 for 0.05–0.1% HCl solution, so the value range of pH was set at 1.0–7.0.

The response surface methodology (RSM) and RCO methodology has been compared for optimization in dehydration of lactobacillus, and the number of proposed experiments by RCO methodology was less than that by RSM [24]. In present study, 12 experiments were conducted based on the 12 sets of proposed factor values given by random search, and 12 extracts were obtained from 12 experiments. Each extract was analyzed for polyphenol content, antioxidant activity, and extract yield, respectively. The values of polyphenol content from 12 extracts were input into RCO program for centroid search, and 4 new sets of proposed factor values were given for polyphenol extraction optimization. It was followed by 4 experiments and obtained 4 extracts. Each extract was analyzed for the polyphenol content again and the values of polyphenol content from 4 extracts were input into RCO program for mapping. The mapping singled out the optimum set of factor values from previous 16 (12 + 4) sets proposed factor values for polyphenol extraction. The final optimum sets of factor values were also obtained for the extraction targets of antioxidant activity and extract yield through the same process of centroid search and mapping. After total 24 (12 + 4 × 3) experiments were conducted, three sets of the optimized factor values were obtained for optimum extraction of polyphenol, antioxidant activity, and extract yield, respectively. This optimization of conditions was obtained in bench scale. It will need more work for an upscaled approach to pilot plant scale.

The RCO program could present three optimized extraction processes for polyphenol content, antioxidant activity and extract yield, which resulted in three corresponding extracts. All three extracts were analyzed for polyphenol content, antioxidant activity and extract yield to confirm their optimum status. The extract obtained from optimum extraction process for polyphenol content has the highest polyphenol content; the extract for yield has the highest extract yield; while the extract for antioxidant activity has the antioxidant activity similar to other two extracts. The extract for polyphenol has been chosen and further analyzed for composition.

There are some inconsistent reports about the composition of Wuweizi fruit. The sugar and protein contents have been analyzed for dried Wuweizi whole fruit, and they are 49~196 mg/g and 10.6~116.9 mg/g, respectively [3,25]. These values are supposed to be higher than that in fresh Wuweizi whole fruit, and even higher than that in the pulp of fresh Wuweizi fruit. It has been reported that the protein and sugar contents of dried wolfberry fruit were higher than fresh wolfberry fruit [26]. In present study, we analyzed sugar, protein, polyphenol and anthocyanin contents in the pulp of fresh Wuweizi fruit. Their components in an order from high to low were sugar (~9.573 mg/g), polyphenol (~1.847 mg/g), protein (~0.327 mg/g) and anthocyanin (0.179 mg/g). The anthocyanin content was analyzed at 0.182~0.189 mg/g for Wuweizi whole fruit [8]. The anthocyanin content in the pulp of fresh Wuweizi fruit is at the same level as anthocyanin content in fresh Wuweizi whole fruit. It indicated that the anthocyanin existed mainly in the pulp of Wuweizi fruit. The polyphenol content in Wuweizi pulp is quite high (~1.847 mg/g) in comparison with that (0.28~0.90 mg/g) in many other fruits, such as apple, pomegranate, banana, grape and londan [27]. Further study would be required in using RCO to optimize polyphenols from other plant sources.

## 3. Experimental Section

### 3.1. Materials

Fresh Wuweizi were purchased in September from Xin Tong agricultural products Company (LiaoYang, Liaoning, China) and kept frozen at −80 °C before use. 1,1-diphenyl-2-picrylhydrazyl (DPPH), Folin-Ciocalteu phenol reagent, and gallic acid were obtained from Sigma-Aldrich Chemical Co. (St. Louis, MO, USA). Bovine serum albumin (BSA) was purchased from Amresco (Solon, OH, USA). Bradford regent was purchased from Bio-Rad Laboratories Inc. (Hercules, California, USA). All other chemicals were of analytical grade and obtained from Beijing Chemical Company (Beijing, China). All solvents were of analytical grade.

### 3.2. Random-Centroid Optimization Process

RCO program was obtained from Dr. Shuryo Nakai of the University of British Columbia. Six factors with a range of values were input into RCO program, including ethanol concentration (0–100%), pH (1–7), liquid and solid ratio (2–10), temperature (30–60 °C), heating time (1–12 h) and extraction times (1–4 times). The random search came out twelve sets of proposed factor values, and each set of proposed factor values was then tested in each experiment. After the twelve experiments, twelve extracts were obtained and analyzed for three extraction target values, including polyphenol content, antioxidant activity, and extract yield. The obtained three extraction target values were input to RCO program for the centroid search, respectively. The four new sets of proposed factor values for each extraction target were presented for further experiments, and the obtained extracts were analysed and the values were input to RCO program again. Finally, the optimum set of factor values was obtained for the extraction target in the form of mapping.

### 3.3. Extraction of Polyphenol From Wuweizi Pulp

3 g pulp puree and ethanol/dH_2_O solution with a set of volumes and ratios were put into 50 mL centrifuge tubes and heated in a waterbath at different temperatures and time in according to the proposed factor values by RCO program. After heating, the mixture was centrifuged at 2810 × g for 3 min. The supernatant was further filtrated by filter paper. The filtrate was concentrated by a vacuum rotary evaporator at 50 °C, and final volume of the concentrate was adjusted to 15 mL with dH_2_O, which was named Wuweizi polyphenol extract (WPE). The WPE was freeze-dried and the dried powder was named WPE freeze-dried powder (WPE-FP). Extract yield (mg/g) is expressed as the weight of WPE-FP (mg) per gram of fresh weight of Wuweizi pulp.

### 3.4. Polyphenol Determination by Folin-Ciocalteu Method

Polyphenol content (PC) of WPE was determined according to the Folin-Ciocalteu method of Singleton & Rossi [28] with some modifications. In 96-well plate, 10 μL of sample solution and 40 μL dH_2_O were mixed thoroughly with 50 μL of Folin–Ciocalteu’s phenol reagent (diluted with dH_2_O of 1:8). After 5 min, 50 μL of 9% (w/v) sodium carbonate was added and mixed immediately. The solution was put in the darkness and at room temperature for 90 min. The absorbance was measured at 630 nm using Microplate reader (M680, Bio-Rad, Japan). Polyphenol content of WPE was estimated from a calibration curve of gallic acid. For the convenience of comparison, the result was further calculated and expressed as milligrams of gallic acid equivalents per gram of fresh weight of Wuweizi pulp: Polyphenol (mg/g) = PC of WPE (mg/mL) × Dilution × 15/3. [15 (mL) is the volume of WPE, 3 (g) is the weight of Wuweizi pulp].

### 3.5. Total Anthocyanin Determination by pH-Differential Method

The content of total anthocyanin was determined by the pH-differential method [29]. The sample was diluted by KCl buffer (0.025 M, pH 1.0) and sodium acetate buffer (0.4 M, pH 4.5), respectively, and then were put in the darkness and at room temperature for 15 min. The absorbances at 510 nm and at 700 nm were measured respectively. The absorbance (A) was calculated as follows: A = (Abs_510nm_ − Abs_700nm_)_pH 1.0_ − (Abs_510nm_ − Abs_700nm_)_pH 4.5_. The total anthocyanin concentration in the original sample was calculated using the following equation: Total anthocyanin (mg/L) = (A × MW × DF × 1000)/(ɛ × L), where MW = 449.2, the molecular weight of Cyanidin 3-*O*-glucoside chloride (Cyd-3-glu); DF, dilution factor; ɛ = 26,900, the molar absorptivity of Cyd-3-glu; L = 1 cm, the path length of cuvette.

### 3.6. Sugar Determination by Phenol-Sulfuric Acid Method

Sugar content of WPE was determined according to Phenol-sulfuric acid method of Dubois *et al.* [30]. 2 mL of sample solution, 1mL of 5% phenol solution, then 5 mL of H_2_SO_4_ were added into 20 mL glass tube. The solution was vortex-mixed thoroughly and put in boiling water bath for 20 min. When the temperature of the solution was down to room temp, the absorbance was recorded at 490 nm using spectrophotometer. Sugar content of WPE was estimated from a calibration curve of glucose. For the convenience of comparison, the result was further calculated and expressed as milligrams of glucose equivalents per gram of fresh weight of Wuweizi pulp.

### 3.7. Protein Determination by Bradford Method

Protein content of WPE was determined according to the method of Bradford [31] with some modifications. In 96-well plate, 160 μL of samples and 50 μL Bradford regent were mixed thoroughly by pipetting and kept in the darkness at room temperature for 90 min. The absorbance was measured at 595 nm using a Microplate reader. Protein content of WPE was estimated from a calibration curve of BSA. For the convenience of comparison, the result was further calculated and expressed as milligrams of BSA equivalents per gram of fresh weight of Wuweizi pulp.

### 3.8. Antioxidant Activity by DPPH Radical Scavenging Method

Antioxidant activity was determined according to the DPPH radical scavenging method of Jing and Kitts [32]. 15 μL of the sample solution, 60 μL of Tris-HCl buffer (0.5 M, pH 7.2), and 150 μL of DPPH ethanol solution (0.15 mM) were added into each well of 96-well plate. The plate was covered with aluminum foil to shield light at room temperature for 30 min. The absorbance was measured at 520 nm using Microplate reader. The DPPH scavenging activity was calculated according to the equation: DPPH scavenging activity scavenging activity (%) = (Abs_control_ − Abs_sample_)/Abs_control_ × 100%.

### 3.9. Statistical Analysis

All experiments were performed in triplicate. The values are presented as mean ± SD. The means were compared by one-way ANOVA, followed by Tukey’s comparisons, using the M SAS 9.1 TS Level 1M3 for Windows (SAS Institute Inc., Cary, NC, USA). The level of confidence required for significance was set at *p* < 0.05.

## 4. Conclusions

RCO methodology is an efficiently global optimization method particularly in investigating multiple factors and targets. The optimum condition for polyphenol extraction from fresh Wuweizi pulp was as follows: 8:1 of liquid and solid ratio (v/w), 67.3% (v/v) of ethanol, 1.75 of initial pH, 55 °C for 4 h, and 4 times. The pH and time were important factors in polyphenol extraction process. The polyphenol extract had high content of polyphenol and anthocyanin along with reasonably good yield and strong antioxidant activity.

## Figures and Tables

**Table 1:** The proposed factor values given by the random search.

Set numbers	Ethanol (%, v/v)	Liquid/ Solid (v/w, mL/g)	pH	Temperature (°C)	Time (h)	Times (times)
1	67.04 (65)	8.87 (9)	3.18	53.71 (55)	3.40 (3)	3.32 (3)
2	10.02 (10)	8.41 (8)	4.32	38.13 (40)	8.96 (9)	1.17 (1)
3	34.19 (35)	3.91 (4)	3.72	46.91 (45)	11.34 (11)	2.41 (2)
4	61.70 (60)	7.28 (7)	1.11	42.62 (45)	10.05 (10)	1.70 (2)
5	26.05 (25)	3.06 (3)	2.92	33.45 (35)	2.93 (3)	2.24 (2)
6	59.56 (60)	3.97 (4)	5.09	53.00 (55)	2.29 (2)	3.61 (4)
7	32.45 (30)	3.37 (3)	1.41	54.36 (55)	7.11 (7)	3.31 (3)
8	40.34 (40)	5.16 (5)	3.63	36.21 (35)	11.74 (12)	2.01 (2)
9	90.13 (90)	5.65 (6)	2.97	52.68 (55)	5.94 (6)	2.11 (2)
10	67.30 (70)	7.93 (8)	1.75	55.32 (55)	4.26 (4)	3.99 (4)
11	62.36 (60)	3.23 (3)	5.41	33.33 (35)	3.44 (3)	2.15 (2)
12	50.61 (50)	3.45 (3)	6.45	39.29 (40)	5.91 (6)	2.47 (2)

“Set numbers” represent various sets of proposed factor values; the values in the table were the proposed values by the RCO program; the values in brackets were the round off values, which were used in following experiments.

**Table 2:** Three extraction target values of the twelve extracts obtained under the proposed extraction conditions.

Extract numbers	Polyphenol (mg/g)	DPPH (%)	Yield (mg/g)
1	1.646 ± 0.008 a,e	50.9 ± 3.4 a	15.58
2	1.463 ± 0.032 f,g	70.1 ± 5.0 d	15.62
3	1.592 ± 0.020 e	75.8 ± 2.0 c,d	16.82
4	1.311 ± 0.022 h	85.8 ± 2.3 e	18.30
5	1.294 ± 0.032 h	68.6 ± 4.2 d	13.60
6	1.671 ± 0.020 a,c	85.8 ± 0.6 e	13.80
7	1.700 ± 0.029 a,b,c	81.7 ± 1.1 b,c,e	14.33
8	1.687 ± 0.009 a,b,c	49.3 ± 5.2 a	15.98
9	1.438 ± 0.020 g	50.6 ± 3.2 a	13.53
10	1.847 ± 0.010 d	85.2 ± 3.6 b,e	16.37
11	1.511 ± 0.004 f	76.8 ± 0.9 b,c,d	13.85
12	1.669 ± 0.015 a,c	83.4 ± 1.3 b,c,e	16.02

“Extract numbers” represent various extracts obtained from proposed extraction conditions; polyphenol and yield were expressed as milligrams per gram of fresh weight of Wuweizi pulp. Values were represented as mean ± SD (*n* = 3).

a–h Means in the same column with different letters represent significant differences (*p* < 0.05).

**Table 3:** The proposed factor values given by centroid search.

Set numbers	Ethanol (%, v/v)	Liquid/ Solid (v/w, mL/g)	pH	Temperature (°C)	Time (h)	Times (times)
**For polyphenol extraction**						
1	47.41 (45)	4.63 (5)	3.68	47.52 (50)	7.11 (7)	2.97 (3)
2	52.88 (55)	5.46 (5)	3.59	48.65 (50)	5.79 (6)	3.12 (3)
3	50.15 (50)	5.53 (6)	3.13	49.92 (50)	6.69 (7)	3.11 (3)
4	51.86 (50)	5.25 (5)	3.60	50.43 (50)	5.72 (6)	3.19 (3)
**For DPPH radical-scavenging activity**						
1	55.66 (55)	4.87 (5)	3.54	46.32 (45)	5.51 (6)	2.87 (3)
2	50.97 (50)	4.99 (5)	3.26	48.58 (50)	6.83 (7)	2.92 (3)
3	55.95 (55)	4.96 (5)	3.92	45.08 (45)	6.22 (6)	2.72 (3)
4	52.93 (50)	4.95 (5)	3.08	47.59 (50)	6.42 (6)	2.86 (3)
**For extract yield**						
1	44.03 (45)	6.02 (6)	3.50	43.08 (40)	8.71 (9)	2.29 (2)
2	53.53 (55)	6.10 (6)	3.31	45.68 (45)	7.78 (8)	2.65 (3)
3	48.48 (50)	6.64 (7)	3.42	46.00 (45)	7.32 (7)	2.51 (3)
4	46.77 (45)	6.93 (7)	2.95	45.48 (45)	8.29 (8)	2.43 (2)

“Set numbers” represent various sets of proposed factor values; the values in the table were the proposed values by the RCO program; the values in brackets were the round off values, which were used in following experiments.

**Table 4:** Extraction target values of each four extracts obtained under proposed extraction conditions.

Extraction targets	Extract numbers			
	1	2	3	4
**Polyphenol (mg/g)**	1.721 ± 0.021 a,b	1.690 ± 0.012 a	1.742 ± 0.003 b	1.717 ± 0.017 a
**DPPH (%)**	84.7 ± 0.9 a	88.9 ± 1.0 b	85.2 ± 2.3 a	81.6 ± 3.3 a
**Yield (mg/g)**	13.87	13.72	13.77	12.83

“Extract numbers” represent 12 extracts obtained from proposed extraction conditions, with 4 extracts obtained and analyzed for each extraction targets. Polyphenol and yield were expressed as milligrams per gram of fresh weight of Wuweizi pulp. Values are represented as mean ± SD (*n* = 3).

a–b Means in the same column with different letters represent significant differences (*p* < 0.05).

**Table 5:** The final proposed optimum factor values for each extraction target given by mapping.

Extraction targets	Ethanol (%, v/v)	Liquid/ Solid (v/w, mL/g)	pH	Temperature (°C)	Time (h)	Times (times)
**Polyphenol**	67.30 (70)	7.93 (8)	1.75	55.32 (55)	4.26 (4)	3.99 (4)
**DPPH**	50.97 (50)	4.99 (5)	3.26	48.58 (50)	6.83 (7)	2.92 (3)
**Yield**	61.70 (60)	7.28 (7)	1.11	42.62 (45)	10.05 (10)	1.70 (2)

The values in the table were the proposed values by the RCO program; the values in brackets were the round off values, which were used in following experiments.

**Table 6:** Extraction target values of three extracts obtained under final optimum extraction conditions.

Extracts	Polyphenol (mg/g)	DPPH (%)	Yield (mg/g)
**Polyphenol**	1.847 ± 0.010 a	85.2 ± 3.6 a	16.37
**DPPH**	0.809 ± 0.019 b	88.9 ± 1.0 a	13.80
**Yield**	1.311 ± 0.022 c	85.8 ± 2.3 a	18.30

Polyphenol content and yield were expressed as milligrams per gram of fresh weight of Wuweizi pulp. Values are represented as mean ± SD (*n* = 3).

a–c Means in the same column with different letters represent significant differences (*p* < 0.05).

**Table 7:** Compositions of three extracts obtained under final optimum extracting conditions.

Extracts	Sugar (mg/g)	Protein (mg/g)	Anthocyanin (mg/g)
**Polyphenol**	9.573 ± 0.068 a	0.327 ± 0.051 a	0.179
**DPPH**	9.397 ± 0.028 b	0.252 ± 0.023 b	0.159
**Yield**	9.367 ± 0.099 b	0.225 ± 0.019 b	0.227

The components were expressed as milligrams per gram of fresh weight of Wuweizi pulp. Values are represented as mean ± SD (*n* = 3).

a–b Means in the same column with different letters represent significant differences (*p* < 0.05).
